# Building trust in the social services through collaboration between child healthcare nurses and parental supporters from the social services, within an extended home-visit programme: a qualitative study

**DOI:** 10.1186/s13104-025-07623-3

**Published:** 2025-12-29

**Authors:** Maria Hjortsjö, Elisabeth Mangrio

**Affiliations:** 1 Department of Social Work, Faculty of Health and Society, Malmö University, Malmö, Sweden; 2 Department of Care Science, Faculty of Health and Society, Malmö University, Malmö, Sweden

**Keywords:** Child healthcare, Collaboration, Home-visit programme, Social services, Trust, Qualitative study

## Abstract

**Objectives:**

One aspect of the Swedish child healthcare is to support parents in their parenting role. The child healthcare nurses are also obligated to report situations that could cause suspicion of violence, and they are encouraged to collaborate with the social services when needed. In the extended home-visit programme called Grow Safely—child healthcare nurses and parental supporters from social service collaborate. There is a need to evaluate how trust could be built between families and the social services through such a collaboration. Thus, the aim of this study was to illuminate—how trust in the social services could be achieved through an extended home-visit programme within the child healthcare in collaboration with parental supporters.

**Results:**

This is a qualitative study and 13 health and social professionals were interviewed. The results showed that it was important to initiate preventive support from the social services at an early stage. It seemed that the child healthcare nurses could build on the high level of trust that they enjoy, as a profession, and that, by introducing the parental supporters from the social services, they enabled the families to start trusting them too.

## Introduction

During the last years, different extended programmes with home visits from the child healthcare have been carried out in Sweden [1–3]. In the county of Scania, the programme, called Grow Safely [4] had a collaborative character, since not only child healthcare nurses have been conducting the visits but also the social services (working as parental supporters), midwives and dental assistants. Grow safely had a preventive focus with the aim of providing support to parents and children and contribute to accessibility to care and equal health within the population in Scania [3]. In order to manage this goal, this intervention differed from the ordinary programme in the extended amount of home visits and the cooperation between the professions that are a part of these visits, which does not exist in the regular child healthcare program [3].

The social services constitute a public service provided by every municipality of Sweden and where social workers possess specialized knowledge about children’s needs [5]. A lot of families receive support from the social services, for instance, if parents have mental health challenges or problems with drug addiction. The social services also provide assistance to families where domestic violence is present [5]. However, these are only examples among a wide range of available services within social service. If domestic violence is present, the health professionals that see and observe it, are, as mentioned above, obligated to report this to the social services. What is reported is a worry about and a concern for the child’s well-being and situation at home, so that the social services are able to support the family in need [5]. In Sweden, professionals who meet children and families—such as those in health care, schools, and social services—are legally obliged under Chap. 19 of the Social Services Act, to immediately report to the social welfare board if they suspect that a child is at risk. This mandatory reporting obligation means that even a suspicion is sufficient to trigger a report; social services then assess the concern and decide whether to initiate an investigation and, if needed, provide protection and support. The Grow Safely program differs from this reactive process by offering early, preventive support before a formal report is made, which emphasizes the preventive focus of this study and illustrates how early interventions can complement mandatory reporting. While mandatory reporting ensures that children at risk receive protection, it also highlights the reactive nature of the system. This makes preventive approaches, such as Grow Safely, particularly important—not only to support families early but also to build trust before a formal report becomes necessary. However, there has, during the last years, been an ongoing discussion about trust in the social services in Sweden and a degree of mistrust has developed, especially among migrants [6, 7].

Trust can be understood as a relational process in which one actor is willing to make themselves vulnerable to the actions of another, based on positive expectations regarding the other’s intentions and behavior. The core of trust lies in the willingness to take risk, rather than the risk itself. Vulnerability implies that something of value may be lost, making trust an active stance rather than a passive trait [8, 9].

In human service work, such as family support practice, this means that a parent who places trust delegates a certain degree of agency to the professional, creating asymmetry and potential vulnerability. Trust is not static; it develops through interaction, communicative acts, and experiences of credibility. It rests on expectations of competence, benevolence, and integrity and is essential for the functioning of the client-professional relationship. Trust is situated and context-dependent; it emerges in the encounter between the profession’s societal mandate and the specific conditions of each client meeting. Thus, trust is both a prerequisite for and an outcome of professional practice [9–11]. Since Grow Safely involved preventive efforts by the social services and not the exercise of public authority, it is of paramount importance to evaluate how trust can be built between families and the social services through the collaboration between child healthcare and social services. Therefore, the aim of this study is to illuminate—based on the perspectives of health professionals and parental supporters—how trust in the social services could be achieved within an extended home-visit programme with collaboration between the child healthcare and parental supporters.

## Main text

### Method

The current qualitative study derives from a larger research project called Grow Safely [3] which ran between 2019 and 2023 and covered both qualitative and quantitative research regarding the experiences of not only the professionals involved but also the parents. The qualitative approach for the current study was needed in order to illuminate experiences [12] of the professionals.

#### Data collection

This current study has been conducted through interviews during 2021 with health, social and dental professionals who have worked within the extended home-visit programme. Six paediatric nurses, one midwife, one dental assistant and five parental supporters responded to the request and participated in the interviews. The informants came from eight different child healthcare centres. This data collection has already resulted in two other publications, one of which centred on the professionals’ experiences of working within this programme and how such collaboration could benefit the professions [13] and the other one on meeting families in different social situations [14]. As the material obtained from the interviews was rich and several research questions were covered, it also included a focus on the professionals’ experiences of establishing trust in the social services and how trust could be achieved within an extended home-visit programme where the child healthcare collaborated with parental supporters. However, since the research question for this current paper was to illuminate how trust in the social services could be achieved within an extended home-visit programme with collaboration between the child healthcare and parental supporters, only meaning units from the interviews with child healthcare nurses and parental supporters were included in this paper and its analysis and child healthcare nurses were the only ones that collaborated with the parental supporters in Grow safely.

The interview guide was created for this specific project and has not been used in earlier studies, see interview guide Table 1. Illuminating these aspects is the aim of the current paper. The interviews lasted between 25 and 76 min.

**Table 1:** Interview guide

How have you overall experienced the home-visits?
Can you tell us about a more positive home-visit?
Can you tell us about a more negative home-visit?
How have the collaboration between you in the team worked out regarding the content within the visits?
How have you experienced the coaching of the teamwork during the home-visits?
How have your team functioned?

#### Data analysis

The data was analysed with inductive thematic content analysis following Burnard [15]. First, the data was read through and coded by the last author. The material that answered the aim of the current study was coded, and the codes that were similar were grouped together into different categories. The category and the sub-categories were derived from the data. Then the coded material was read by the first author, who provided feedback on it. See the analysis process in Table 2.

**Table 2:** Analysis schedule—from meaning unit to code to sub-category

Meaning unit	Code	Sub-category
We work to create trust and confidence towards us and […] we downplay the social services, which feels very important to do right now.	Establish a setting together with the social services aimed at de-dramatising their involvement and strengthening trust.	Reduce fear of the social services
And the benefits of us, who are not a family centre, having established increased collaboration—for we have talked about this for a long time—are that we need to cooperate with parental supporters, which is not the same thing as collaborating with the social services. This is because we see that we have a healthcare centre in an area with a high CNI, right? We have many families in need of support. And in that context, having this collaboration is extremely valuable—not only for us, as it helps us build a relationship with them, but also because it allows families to receive help at an early stage. (Informant 3)	Good with collaboration with the parental supporters in order for the families to be able to access support earlier, when needed.	Provide early preventive support

### Results

The analysis of the results resulted in one overarching category: *trust is built through listening and collaboration and* four different sub-categories: *Listen to and be engaged in the families*,* Provide early preventive support*,* Reduce fear of the social services* and *How the collaboration between professionals reassured the parents.*

#### Trust is built through listening and collaboration

The results showed that building trust and confidence in families was done through active listening, early preventive support, and collaborative professional efforts that reduce fear and foster reassurance. But also through building trust through first the child healthcare nurses and then after the child healthcare nurses introduced the parental supporters, the trust was transferred over to the parental supporters, which could be described through a chain of trust.

#### Listen to and be engaged in the families

The professionals talked about the families that they met during the home visits and how they sometimes met with families that had, prior to the visit, got a report of concern from the social services and how this affected the way they tried to interact with the family. They stressed the importance of listening to the family and being attuned to where the family was emotionally and then start from there. One professional said this:

*“It’s important to sense where the family is at and then be sensitive and listen to them and take the planned topics for the visit when we can feel that they are ready for it”.* (Informant: parental supporter 9)

Another professional said that during the programme they had time to build trust in the social services through their engagement and the visits they made to the families. Yet another professional mentioned that sometimes they met with families who had had relatives who had been in contact with the social services, or who had themselves grown up with parents that had had contact with the social services. The professional emphasised how important this programme is for those families, in that it offers a valuable opportunity to build trust, and to listen to the concerns the families may have regarding their children and to their fears of being judged or misunderstood by the authorities. Overall, the professionals highlighted how clearly parents express their desire for their children to grow up in a safe and socially stable environment—free from the challenges they themselves have experienced.

#### Provide early preventive support

The professionals talked about how, through this programme, they were able to offer support at an early stage to parents. When the child healthcare and the social services work closely together in this way, it becomes possible to introduce one another to the families and create space for continued contact even after the programme has ended. Families may be in need of advice and support at an early stage, in order to prevent problems and difficulties from escalating and becoming more complex. One professional said:

*“This is very good for us that are not located within a family centre*,* to be able to collaborate closely with the social services*,* since we have many families in need of support*,* and through this programme they can get early support and help from the social services”. (Informant: paediatric nurse 3)*

Another child healthcare nurse stressed that one of the points of this programme was that they could offer support for families in need of support from the social services and, not least, that this support could be offered together with the family instead of making a report of concern to the social services.

#### Reduce fear of the social services

The professionals mentioned how they considered that the work within Grow Safely could normalise the presence of the social services. The fact that, during the home visits, the parental supporters from the social services come together with the child healthcare nurse, instead of parental supporters coming on their own from the social services, makes their presence less threatening. One professional said the following:

“*We want to downplay the threatening part of us coming from the social services and say as it is*,* but that they [the parents]*,* through all the meetings with us*,* should have a chance of understanding that the social services are much more than taking away children from their parents but offer support and help in their parenting. There’s a great range in what we do within our work*,* it’s important that the parents understand that*”. (Informant: parental supporter 4)

Since the parental supporters informed the parents that they were from the social services, they could sense that some parents probably said no thanks to the programme because they were hesitant to be in contact with the social services through the parental supporters. The parental supporters nevertheless thought it was worth telling the parents about their connection with the social services, since it might mean that the families would gain more trust in the social services through meeting the parental supporters. They also believed that they could easily transfer the trust that the families usually have in the child healthcare to the social services and thereby downplay the potentially threatening aspect of the latter. But one of the parental supporters concluded that the fact that they are employed by the social services is nevertheless a stumbling block.

#### How the collaboration between professionals reassured the parents

The professionals recounted that the midwife made the first home visit together with the child healthcare nurse, and that the nurse made the second visit together with the parental supporter and introduced the parental supporter to the family, continuing the dialogue from the first visit. Hence, the parental supporter was introduced to the family in a smooth way and trust could be built between the different professionals within this programme. One nurse expressed it like this:

“*Of course*,* I kind of own the contact with the family since I was there during the first visit. But since I then also*,* by now*,* know the parental supporters very well and we are getting used to working together and kind of having a dialogue with families during our visits*,* it gets quite easy to introduce the parental supporter to the family and also get the family to trust the parental supporter*”. (Informant: paediatric nurse 3)

One of the parental supporters highlighted a key benefit of the programme, namely, the fact that she and the nurse she worked with had become more visible to one another, recognising and appreciating each other’s roles and contributions. Through this closer contact, they are able to work together to meet the needs of the families and to build trust with the families.

### Discussion

The results showed that building trust and confidence in families was done through active listening, early preventive support, and collaborative professional efforts that reduce fear and foster reassurance. Building trust first through the child healthcare nurses and then after that the trust was transferred over to the parental supporters through the child healthcare nurses. It seemed that the child healthcare nurses could build on the high degree of trust that they enjoy as a profession, and that they could, through introducing the parental supporters, enable the families to start trusting them too. The trust-building part of extended home-visit programs has also been seen in an earlier study among parents receiving such a program [16]. Another Swedish study with the perspectives of parents participating in these programs, lifted that the parents they felt free to ask for extended professional support, such as extra appointments with parental supporters working within social services [17], which could be understood as trust was built. Also other Swedish studies on the professionals perspectives, perceived that parents felt more secure and willing to contact social services when needed [18] and they could arrange such meetings easier through this collaboration compared with before [19].

Building trust in the social services is of great importance, especially today, since rumours have been spreading about the social services taking children away from their parents [6, 7], rumours that have mainly concerned migrant families in Sweden. The social services have a wide range of responsibilities and one of those responsibilities is to support and help parents in their parenting, which is why it is important that all families with children get a good impression of and feel trust in the social services, in case they will need support in the future. In conclusion, the findings demonstrate that trust is a fundamental prerequisite for effective preventive family support, emphasizing the need to institutionalize trust-building strategies through collaboration between child healthcare and social services. Future research should deepen understanding of how these mechanisms function across cultural contexts and their long-term impact on families’ willingness to seek support.

### Conclusions

Trust was built by the professionals being able to listen to the families and to meet the families where they were, trying to build trust from there. But it was also crucial to initiate preventive support from the social services at an early stage, in families whose situation risked becoming more difficult. Preventive work around families in Sweden has to consider the importance of trust in the social services, as the trust level towards social care has been challenged and as trust is fundamental in order to be able to reach families in need.

### Limitations

In total, 13 professionals were interviewed. The interviews were quite long and they were rich in data and met saturation. The analysis of the data for the present study was first done by the first author and then checked and validated by the second author, which improves the credibility of the analysis [12]. However, it could be considered a limitation that the transcripts were not returned to the informants after the interviews were done. Another limitation is that the study relies solely on professionals’ perspectives, which aligns with its focus on professional practice, but future research should incorporate families’ voices to provide a more comprehensive understanding of trust-building.

## Data Availability

The datasets generated or analysed during the current study are available from the corresponding author on reasonable request.
